# Nationwide study of spondyloarthritis spectrum and disease characteristics in Egypt

**DOI:** 10.1038/s41598-025-15046-0

**Published:** 2025-08-13

**Authors:** Yasser Emad, Ahmed Elsaman, Hanan El-Saadany, Rawhya R. ElShereef, Yousra Hisham, AlShaymaa Farouk, Gehad G. Elsehrawy, Samar Tharwat, Marwa A. Amer, Osman Hammam, Nevin Hammam, Amany S. ElBahnasawy, Rasha Fawzy, Fatemah Elshabacy, Asmaa Khalifa, Abdelhfeez Moshrif, Faten Ismail, Shereen Elwan, Mahmoud A. Abdelsalam, Amira M. Ibrahim, Tamer A. Gheita

**Affiliations:** 1 Rheumatology Department, Faculty of Medicine, Cairo University, Cairo, Egypt; 2 Rheumatology Department, Faculty of Medicine, Sohag University, Sohag, Egypt; 3 Rheumatology Department, Faculty of Medicine, Tanta University, Gharbia, Egypt; 4 Rheumatology Department, Faculty of Medicine, Minia University, Minia, Egypt; 5 Rheumatology Department, Faculty of Medicine, Alexandria University, Alexandria, Egypt; 6 Internal Medicine Department, Rheumatology unit, Faculty of Medicine, Ain Shams university, Cairo, Egypt; 7 Rheumatology Department, Faculty of Medicine, Suez Canal University, Ismailia, Egypt; 8 Internal Medicine Department, Rheumatology unit, Faculty of Medicine, Mansoura university, El Gomhouria St, Mansoura, Dakahlia, Egypt; 9 Department of Internal Medicine, Faculty of Medicine, Horus University, New Damietta, Egypt; 10 Rheumatology Department, Faculty of Medicine, New Vally University, New Valley, Egypt; 11 Rheumatology Department, Faculty of Medicine, Assuit University, Assuit, Egypt; 12 Rheumatology Department, Faculty of Medicine, Mansoura university, Dakahlia, Egypt; 13 Rheumatology Department, Faculty of Medicine, Benha University, Benha, Kalyoubia Egypt; 14 Rheumatology Department, Benha Teaching Hospital, Benha, Kalyoubia Egypt; 15 Rheumatology Department, Faculty of Medicine, Al-Azhar University, Assuit, Egypt; 16 Rheumatology Department, Faculty of Medicine, Kafr ElSheikh University, Kafr ElSheikh, Egypt

**Keywords:** Spondyloarthritis, Age at onset, Gender, Biologics, Egypt, Multicenter, Musculoskeletal system, Rheumatic diseases, Rheumatology

## Abstract

The aim of this study was to describe the array and disease characteristics of spondyloarthritis (SpA) across Egypt. This work included 1401 SpA patients recruited from 15 specialized Egyptian rheumatology centers representing 20 major cities. The demographic and clinical features, as well as the therapeutic data, were recorded. The mean age of the patients was 37.6 ± 11.4 years, disease duration 8.01 ± 6.7 years, and age at onset 29.9 ± 11 years; 148 (10.6%) were juvenile-onset. There were 813 males and 588 females (M: F 1.4:1). 5.7% were diabetic, 6.1% hypertensive, and 19.3% were smokers. The mean BASDAI was 3.98 ± 1.78, and the BASFI was 4.02 ± 1.77. The human leukocytic antigen (HLA-B27) was positive in 19.8%. Biologic therapy was received by 55.5%, followed by methotrexate (36%) ,steroids (10.8%), and sulfasalazine in 10.7%. In males, the age at onset was significantly lower (p = 0.02), while radiographic axSpA, neuropsychiatric and pulmonary manifestations, HLA-B27 positivity, and receiving biologic therapy were significantly higher (p = 0.02, p < 0.0001, p = 0.03, p < 0.0001, and p < 0.0001). In females, cutaneous manifestations and arthritis were significantly more frequent (p < 0.0001 and p < 0.0001). Those with positive HLA-B27 had a significantly higher frequency of AS (73%) (p = 0.003), male gender (66.2%) (p < 0.0001), longer disease duration (p = 0.001), and were receiving a higher frequency of biologic therapy (89%, p < 0.0001). Radiographic axial SpA was most reported from Assuit (15.9%), nr-axSpA from Cairo (24.5%), peripheral arthritis only from Giza (30.7%), and unclassified from Kafr ElSheikh (33.9%) (p = 0.002). The spectrum of SpA in Egypt is inconsistent across the country. Gender, disease subtype, and HLA-B27 seem to play a key role in the phenotypic presentation.

## Introduction

A group of related chronic inflammatory diseases with phenotypically distinct disorders are subsumed as ‘spondyloarthritis’ (SpA). They share common features involving the axial skeleton (sacroiliac joints ‘SIJs’ and spine), dactylitis, enthesitis, and asymmetric mono- or oligoarthritis with certain extra-articular manifestations such as anterior uveitis, psoriasis (PSO), and inflammatory bowel disease (IBD), as well as an association with the human leucocyte antigen-B27 (HLA-B27)^1^. The SpA group includes ankylosing spondylitis (AS), reactive arthritis (ReA), psoriatic arthritis (PsA), arthritis associated with IBD, and a subgroup of juvenile idiopathic arthritis (JIA)^2^.

Depending on the leading manifestation, two major SpA groups are defined: axial SpA (axSpA), mainly involving the SIJs and/or spine, and peripheral SpA, with predominant arthritis, enthesitis, and dactylitis^3^. AxSpA is characterized by inflammatory back pain (IBP) and has replaced the long-used term AS. The SIJ is frequently initially affected, and structural changes of the spine add at later stages^4^. Based on the latter, two subgroups of axSpA are based on the presence of structural alterations in the SIJs: radiographic (r)-axSpA and non-radiographic (nr)-axSpA^5^. IBP poses as the hallmark symptom in axSpA^6^. Considerable evidence indicates the developmental pathways crucially involved in osteoimmunology as a mechanism underlying the development of bone damage in the context of SpA^7,8^.

AS, the most prevalent and prototypical subtype of SpA, predominantly affects the axial skeleton and is classically defined by radiographic sacroiliitis according to the modified New York criteria. AS typically presents at a young age, with initial symptoms arising in the second to third decade of life, and it is uncommon for disease onset to occur after the age of 45. Nevertheless, a minority of patients—approximately 3.5–13.8%—may experience late-onset AS (LoAS), which can pose diagnostic challenges due to its atypical presentation^9^.

Diagnosis of axSpA relies on clinical features, laboratory tests, and imaging, particularly MRI, and management includes exercise, lifestyle changes, non-steroidal anti-inflammatory drugs (NSAIDs), and biological and targeted-synthetic disease-modifying anti-rheumatic drugs (DMARDs)^10^. Comorbidities exert distinct influences on disease activity, physical function, and health-related quality of life in male and female patients with SpA^11^.

The disease activity is usually monitored using validated measures such as the Bath AS disease activity index (BASDAI) or the AS disease severity index (ASDAS), which are recommended for a better outcome^12^. Physical function is an important determinant of health-related QoL in radiographic axial SpA patients (r-axSpA)^13^.

Reliable interpretation of imaging findings is essential for the diagnosis of axSpA^14^ and radiographic sacroiliitis is a key criterion for classification. With the availability of MRI of the SIJ, early findings could point to the disease before definite changes become visible on radiographs^15^. In confirmed r-axSpA, ~ 40% of patients are originally considered to have nr-axSpA. The distinction between r-axSpA and nr-axSpA may be ill-defined in routine clinical practice^16^.

Despite growing recognition of SpA’s global heterogeneity, large-scale data from North Africa, particularly Egypt, remain scarce. Critical gaps persist in understanding regional variations in HLA-B27 prevalence, gender disparities, and phenotypic patterns, which are pivotal for early diagnosis and tailored treatment. Existing studies in Egypt are limited by small sample sizes or single-center designs^17–19^leaving questions about nationwide disease characteristics unanswered. This study addresses these gaps by providing the first comprehensive, multicenter analysis of SpA across Egypt, elucidating demographic, clinical, and geographic influences on disease presentation.

### Patients and methods

This observational cross-sectional study was conducted encompassing axSpA patients from 15 specialized rheumatology centers across Egypt, representing 20 major cities. Diagnostic classification adhered to established criteria: Assessment of SpondyloArthritis International Society (ASAS) criteria^20^ for axial SpA (axSpA, including radiographic and non-radiographic subtypes), Classification Criteria for Psoriatic Arthritis (CASPAR) for PsA, European Spondyloarthropathy Study Group (ESSG) criteria for reactive arthritis (ReA) and enteropathic arthritis (EnA), and International League of Associations for Rheumatology (ILAR) criteria for juvenile SpA (JSpA). Undifferentiated SpA was diagnosed when clinical features aligned with SpA but did not meet criteria for specific subtypes. A steering committee resolved diagnostic uncertainties to ensure consistency across centers. Exclusions included pregnancy, concomitant rheumatological conditions, and non-cooperative participants. Prior to inclusion, all participants were thoroughly briefed on the study’s objectives and procedures. Ethical committee approval was obtained from the Suez Canal University Research Ethics Committee (Approval No: 5459#) before the commencement of the work. Patients were consecutively recruited from outpatient clinics between January 2020 and December 2023 to ensure a representative sample and minimize selection bias.

Participants underwent comprehensive evaluations, including detailed medical histories, clinical examinations, assessments of disease activity and functional status using BASDAI and Bath AS functional index (BASFI)^21^scores, as well as a complete laboratory profile. X-ray and/or MRI imaging were utilized for diagnostic purposes. Medications encompassing NSAIDs, biologics, and anti-ILs were documented.

### Assessment of fibromyalgia and extra-articular features

Fibromyalgia was diagnosed using the 2016 ACR criteria^22^requiring chronic widespread pain and symptom severity scoring. Neuropsychiatric manifestations (e.g., depression, neuropathy) were evaluated via clinical assessment and corroborated with imaging (MRI/CT) or specialist consultation when indicated. Pulmonary involvement was identified through clinical symptoms (e.g., dyspnea) and confirmed by pulmonary function tests or imaging (chest X-ray/HRCT) as needed.

### Recruitment and data harmonization

To ensure data standardization, all investigators underwent centralized training on the study protocol, including the application of ASAS criteria, clinical scale administration (BASDAI, BASFI), and imaging interpretation. Diagnostic consistency was maintained through regular case discussions and audits. Data were collected using standardized forms and digitized to minimize inter-center variability. Additionally, a steering committee reviewed ambiguous cases to confirm eligibility.

### Statistical analysis

Statistical Package for Social Science (SPSS) version 22 was used. Data was presented as mean ± SD (range), number, and frequency (%). The Mann-Whitney U test and Kruskal-Wallis test were used for comparison of samples. Results were adapted for missing values. Significance was set at *p* < 0.05.

## Results

The mean age of the patients was 37.6 ± 11.4 years, disease duration 8.01 ± 6.7 years, and 2% were juvenile-onset. The M: F was 1.4:1. Cases were more frequently presented from Kafr El-Sheikh (13.6%), Cairo (11.3%), Assuit (10.6%), Giza (9.6%), Alexandria (9.4%), Sohag (7.9%), Gharbia (7.4%), Minia (7.2%), Mansoura (6.2%), Ismailia (4.5%), and Kalyoubia (3.9%) and minor frequencies from governorates across Egypt, including Menoufiya, Beheira, Port said, Suez, Sharkia, BeniSuef, Aswan, and Qena. 63.3% were married, and abortion was reported in 1.4% (*n* = 20). 5.7% were diabetic, 6.1% hypertensive, and 19.3% were smokers. Characteristics according to gender are presented in Table 1. Only 7 cases were children. Arthralgias were present in 36.7%. 48.8% were active, 50.6% had low activity, and 0.6% were in remission.

**Table 1:** Demographics, spectrum, clinical, laboratory characteristics of the spondyloarthritis patients according to the gender.

	Characteristic mean ± SD or *n*(%)	SpA (*n* = 1401)	Male (*n* = 813)	Female (*n* = 588)	*P*
Demographic	Age (years)	37.6 ± 11.4	37.7 ± 11.6	37.4 ± 11.2	0.62
	Disease duration (years)	8.01 ± 6.7	8.7 ± 7.04	7.03 ± 6.1	**< 0.0001**
	Age at onset (years)	29.9 ± 11	29.3 ± 10.8	30.7 ± 11.1	**0.02**
	Family Hx of RD	52 (3.7)	33 (4.1)	19 (3.2)	0.28
Feature	r-axSpA	695 (49.6)	440 (54.1)	255 (43.4)	**0.03**
	nr-axSpA	267 (19.1)	149 (18.3)	118 (20.1)	
	Periph. Arthritis only	129 (9.2)	78 (9.6)	51 (8.7)	
	Unclassified	310 (22.1)	145 (17.8)	161 (27.4)	
Subtype	Ankylosing spondylitis	599 (42.8)	408 (50.2)	191 (32.5)	0.07
	Psoriatic arthritis	364 (26)	162 (19.9)	202 (34.4)	
	Enteropathic arthritis	34 (2.4)	12 (1.5)	22 (3.7)	
	Reactive arthritis	39 (2.8)	11 (1.4)	28 (4.8)	
	Undifferetiated SpA	217 (15.5)	133 (16.4)	84 (14.3)	
	Juvenile SpA	148 (10.6)	87 (10.7)	61 (10.4)	
Manifestations	iLBP	303 (21.6)	199 (24.5)	104 (17.7)	0.32
	Cutaneous	307 (21.9)	135 (16.6)	172 (29.3)	**< 0.0001**
	Oral ulcers	17 (1.2)	10 (1.2)	7 (1.2)	0.9
	Arthritis	740 (52.8)	363 (44.6)	377 (64.1)	**< 0.0001**
	Dactylitis	40 (2.9)	24 (3)	16 (2.7)	**0.001**
	Enthesitis	209 (14.9)	139 (17.1)	70 (11.9)	0.15
	Ocular	129 (9.2)	84 (10.3)	45 (7.7)	0.47
	Neuropsychiatric	37 (2.6)	31 (3.8)	6 (1)	**< 0.0001**
	Gastrointestinal	95 (6.8)	59 (7.3)	36 (6.1)	0.51
	Cardiovascular	26 (1.9)	13 (1.6)	13 (2.2)	0.52
	Pulmonary	66 (4.7)	46 (5.7)	20 (3.4)	**0.03**
	Renal	21 (1.5)	14 (1.7)	7 (1.2)	0.32
	Fibromyalgia	54 (3.9)	26 (3.2)	28 (4.8)	0.88
BASDAI		3.98 ± 1.78	3.93 ± 1.73	4.1 ± 1.8	0.31
BASFI		4.02 ± 1.77	4.13 ± 1.8	3.8 ± 1.7	0.07
Laboratory	Hemoglobin (g/dl)	12.2 ± 1.76	12.4 ± 1.66	11.9 ± 1.85	**< 0.0001**
	TLC (x10^3^/mm^3^)	7.32 ± 3.48	7.38 ± 3.86	7.24 ± 2.94	0.52
	Platelets (x10^3^/mm^3^)	279.3 ± 95	285.7 ± 95.8	271.2 ± 93.6	**0.015**
	ESR (mm/1st h)	36.4 ± 23.7	35.5 ± 24.1	37.7 ± 23.1	0.12
	ALT(U/L)	24.9 ± 13.2	24.3 ± 11.2	25.7 ± 15.2	0.14
	Creatinine (mg/dl)	1.45 ± 6.8	1.45 ± 6.8	1.45 ± 6.8	0.31
	SUA (mg/dl)	4.5 ± 2.03	4.4 ± 2.2	4.6 ± 1.9	0.53
	CRP (mg/dl) (+ ve 615)	14.6 ± 17.8	14.4 ± 16.8	14.9 ± 19.1	0.7
	HLA-B27	278 (19.8)	184 (22.6)	94 (16)	**< 0.0001**
Sacroiliitis –x-ray MRI		221 (15.8) 497 (35.5)	143 (17.6) 310 (38.1)	78 (13.3) 187 (31.8)	0.58 0.88
Enthesitis		209 (14.9)	139 (17.1)	70 (11.9)	0.15
Medications	NSAIDs	197 (14.1)	107 (13.1)	90 (15.3)	0.26
	Steroids	152 (10.8)	88 (10.8)	64 (10.9)	0.12
	Hydroxychloroquine	37 (2.6)	19 (2.3)	18 (3.1)	0.34
	Azathioprine	24 (1.7)	12 (1.5)	12 (2)	0.39
	Cyclosporin A	30 (2.1)	18 (2.2)	12 (2)	0.56
	Methotrexate	505 (36)	242 (29.8)	263 (44.7)	**< 0.0001**
	Leflunomide	112 (8)	62 (7.6)	50 (8.5)	0.32
	Sulfasalazine	150 (10.7)	86 (10.6)	64 (10.9)	**0.001**
	Biologic	777 (55.5)	464 (57.1)	313 (53.2)	**< 0.0001**

SpA: spondyloarthritis, RD: rheumatic disease, r-axSpA: radiographic axial SpA, nr-axSpA: non-radiographic axSpA, iLBP: inflammatory low back pain, BASDAI: Bath ankylosing spondylitis disease activity index, BASFI: bath AS functional index, TLC: total leucocytic count, ESR: erythrocyte sedimentation rate, ALT: alanine transaminase, SUA: serum uric acid, CRP: C-reactive protein, HLA-B27 human leucocytic antigen B27, MRI: magnetic resonance imaging. Bold values are significant at *p* < 0.05.

Regarding medications, 197 (14.1%) were regularly using NSAIDs, 18 (1.3%) were receiving low-dose aspirin, and 1.3% receiving colchicine. Biologic therapy was received by 777 patients and included secukinumab (34.4%), adalimumab (31.9%), etanercept (20.5%), golimumab (15.2%), infliximab (3.1%), certolizumab (0.8%), ixekizumab (0.5%), ustekinumab (0.4%), tocilizumab (0.3%), and tofacitinib (0.13%). 3.5% of those receiving biologics had received 3 types, while 7.3% received 2 types.

Differences in the features according to the disease subtypes are presented in Table 2. Those with positive HLA-B27 had a significantly higher frequency of AS (73%) (*p* = 0.003), male gender (66.2%) (*p* < 0.0001), longer disease duration (9.3 ± 7.5 years vs. 7.02 ± 6.7) (*p* = 0.001), and were receiving a higher frequency of biologic therapy (89%) (*p* < 0.0001). Lower frequency of arthralgia (34.8%) (*p* < 0.0001), arthritis (38.8%) (*p* < 0.0001), steroid intake (5.2%) (*p* = 0.005), and MTX use (23.6%) (*p* < 0.0001).

**Table 2:** Demographics, spectrum, clinical, laboratory characteristics of the spondyloarthritis patients according to the subtypes.

	Characteristic mean ± SD or *n*(%)	AS (599)	PsA (364)	EnA (34)	ReA (39)	UnSpA (217)	JSpA (148)	*p*
Demographic	Age (y)	39.1 ± 10	42.4 ± 11.6	38.4 ± 10.6	37.8 ± 11.4	37.6 ± 10.7	24.6 ± 8	**< 0.0001**
	Disease duration (y)	9.5 ± 7.1	6.04 ± 5.7	3.3 ± 2.6	3.2 ± 2.5	7 ± 5.2	10.6 ± 8	**< 0.0001**
	Age at onset (y)	29.7 ± 8.1	36.5 ± 11.3	35.1 ± 10.3	34.7 ± 10.3	28.4 ± 8.3	14 ± 3.04	**< 0.0001**
	Gender M: F	2.1:1	0.8:1	0.54:1	0.4:1	1.6:1	1.4:1	**< 0.0001**
	Family Hx of RD	15 (2.5)	13 (3.6)	0 (0)	0 (0)	18 (8.3)	6 (4.1)	**0.03**
Feature	r-AxSpA	439 (73.2)	65 (17.8)	14 (41.2)	26 (66.6)	63 (29.1)	92 (62.2)	**< 0.0001**
	Nr-AxSpA	56 (9.3)	43 (11.8)	13 (38.2)	6 (15.4)	130 (59.9)	19 (12.8)	
	Periph.Arthritis only	18 (3)	80 (21.9)	1 (2.9)	4 (10.3)	9 (4.1)	17 (11.5)	
	Unclassified	86 (14.4)	176 (48.4)	6 (17.6)	3 (7.7)	15 (6.9)	20 (18.5)	
Manifestations	iLBP	132 (22)	21 (5.7)	2 (5.8)	1 (2.6)	101 (46.5)	46 (31.1)	0.36
	Cutaneous	6 (1)	277 (76.1)	1 (2.9)	0 (0)	6 (2.7)	17 (11.5)	**< 0.0001**
	Oral ulcers	8 (1.3)	6 (1.6)	0 (0)	0 (0)	2 (0.9)	1 (0.7)	0.37
	Arthritis	205 (34.2)	293 (80.5)	26 (76.5)	39 (100)	97 (44.7)	80 (54.1)	**< 0.0001**
	Dactylitis	3 (0.5)	29 (7.9)	0 (0)	0 (0)	3 (1.4)	5 (3.4)	0.35
	Enthesitis	86 (14.4)	33 (9.1)	0 (0)	1 (2.6)	49 (22.6)	40 (27.02)	**< 0.0001**
	Ocular	60 (10)	18 (4.9)	2 (5.8)	2 (5.1)	27 (12.4)	20 (18.5)	**0.03**
	Neuropsychiatric	4 (0.6)	2 (0.5)	1 (2.9)	0 (0)	27 (12.4)	3 (2)	**< 0.0001**
	Gastrointestinal	43 (7.2)	9 (2.5)	14 (41.2)	2 (5.1)	18 (8.3)	9 (6.1)	**< 0.0001**
	Cardiovascular	5 (0.8)	7 (1.9)	0 (0)	0 (0)	11 (5.1)	3 (2)	**0.003**
	Pulmonary	30 (5)	9 (2.5)	1 (2.9)	3 (7.7)	17 (7.8)	6 (4.1)	0.11
	Renal	5 (0.8)	3 (0.8)	0 (0)	1 (2.6)	11 (5.1)	1 (0.7)	**< 0.0001**
	Fibromyalgia	16 (2.7)	27 (7.4)	1 (2.9)	0 (0)	8 (3.5)	2 (1.4)	**0.03**
BASDAI		4.1 ± 1.7	4.6 ± 1.9	3.6 ± 1.5	3.7 ± 1.1	3.2 ± 1.6	4 ± 1.8	**< 0.0001**
BASFI		4.3 ± 1.7	2.8 ± 1.1	5.9 ± 5	0.9 ± 0.9	3.7 ± 1.8	4.1 ± 1.6	**0.001**
Laboratory	Hemoglobin (g/dl)	12.5 ± 1.7	12 ± 1.5	10.3 ± 2.5	10.9 ± 1.5	12.4 ± 1.6	12.2 ± 2	**< 0.0001**
	TLC (x10/mm3)	7.4 ± 3.9	7.12 ± 4	8 ± 2.8	6.9 ± 2.5	7.4 ± 2.3	7.3 ± 3.5	0.71
	Platelets (x10/mm3)	281.7 ± 98	263.5 ± 88.4	260.6 ± 86.1	220.3 ± 89.2	301.2 ± 84.8	279.3 ± 95	**< 0.0001**
	ESR (mm/1st h)	34.4 ± 22.9	38.7 ± 20.3	43.5 ± 27.6	35.4 ± 12.4	36.6 ± 23.3	38.4 ± 32.7	0.1
	ALT (U/L)	25.7 ± 11.4	24.6 ± 10.1	23.7 ± 10.1	31.7 ± 18.8	23.6 ± 22.1	21.7 ± 11.7	**0.002**
	Creatinine (mg/dl)	1.3 ± 5.3	0.9 ± 0.33	0.8 ± 0.2	0.8 ± 0.2	2 ± 11.8	3.6 ± 13.8	0.07
	SUA (mg/dl)	4.5 ± 1.9	4.8 ± 1.7	4.6 ± 2.2	4.8 ± 1.4	3.6 ± 3	3.2 ± 2.8	**0.005**
	CRP (mg/dl)	15.6 ± 18.6	11.2 ± 14.1	20.9 ± 28.3	8.3 ± 9.5	17.1 ± 16.7	14.6 ± 20.8	**< 0.0001**
	HLA-B27	203 (33.9)	4 (1.1)	5 (14.7)	9 (23.1)	33 (15.2)	24 (16.2)	**< 0.0001**
Sacroiliitis –x-ray MRI		124 (20.7) 260 (43.4)	20 (5.5) 53 (14.6)	4 (11.8) 15 (44.1)	0 (0) 31 (79.5)	51 (23.5) 83 (38.2)	22 (14.9) 55 (37.2)	**0.006** **0.002**
Enthesitis		86 (14.4)	33 (9.1)	0 (0)	1 (2.6)	49 (22.6)	40 (27.1)	**< 0.0001**
Medications	Steroids	38 (6.3)	52 (14.3)	12 (35.3)	5 (12.8)	25 (11.5)	20 (13.5)	**< 0.0001**
	Hydroxychloroquine	5 (0.8)	16 (4.4)	1 (2.9)	2 (5.1)	4 (1.8)	9 (6.1)	**< 0.0001**
	Azathioprine	4 (0.7)	7 (1.9)	7 (20.6)	0 (0)	3 (1.4)	3 (2.1)	**< 0.0001**
	Cyclosporin A	6 (1)	12 (3.3)	1 (2.9)	0 (0)	5 (2.3)	6 (4.1)	**< 0.0001**
	Methotrexate	96 (16.1)	273 (75)	15 (44.1)	27 (69.3)	39 (18)	55 (37.2)	**< 0.0001**
	Leflunomide	22 (3.7)	65 (17.9)	2 (5.9)	3 (7.7)	8 (8.3)	12 (8.1)	**0.001**
	Sulfasalazine	51 (8.5)	48 (13.2)	4 (11.8)	3 (7.7)	26 (12)	18 (12.2)	**< 0.0001**
	Biologic	389 (64.9)	140 (38.5)	16 (47.1)	8 (20.5)	152 (70.1)	72 (48.6)	**< 0.0001**

SpA: spondyloarthritis, AS: ankylosing spondylitis, PsA: psoriatic arthritis, EnA; enteropathic arthritis, ReA: reactive arthritis, UnSpA: undifferentiated SpA, JSpA: juvenile-onset SpA, RD: rheumatic disease, r-axSpA: radiographic axial SpA, nr-axSpA: non-radiographic axSpA, iLBP: inflammatory low back pain, BASDAI: Bath AS disease activity index, BASFI: bath AS functional index, TLC: total leucocytic count, ESR: erythrocyte sedimentation rate, ALT: alanine transaminase, SUA: serum uric acid, CRP: C-reactive protein, HLA-B27 human leucocytic antigen B27, MRI: magnetic resonance imaging. Bold values are significant at *p* < 0.05.

**Fig. 1:**
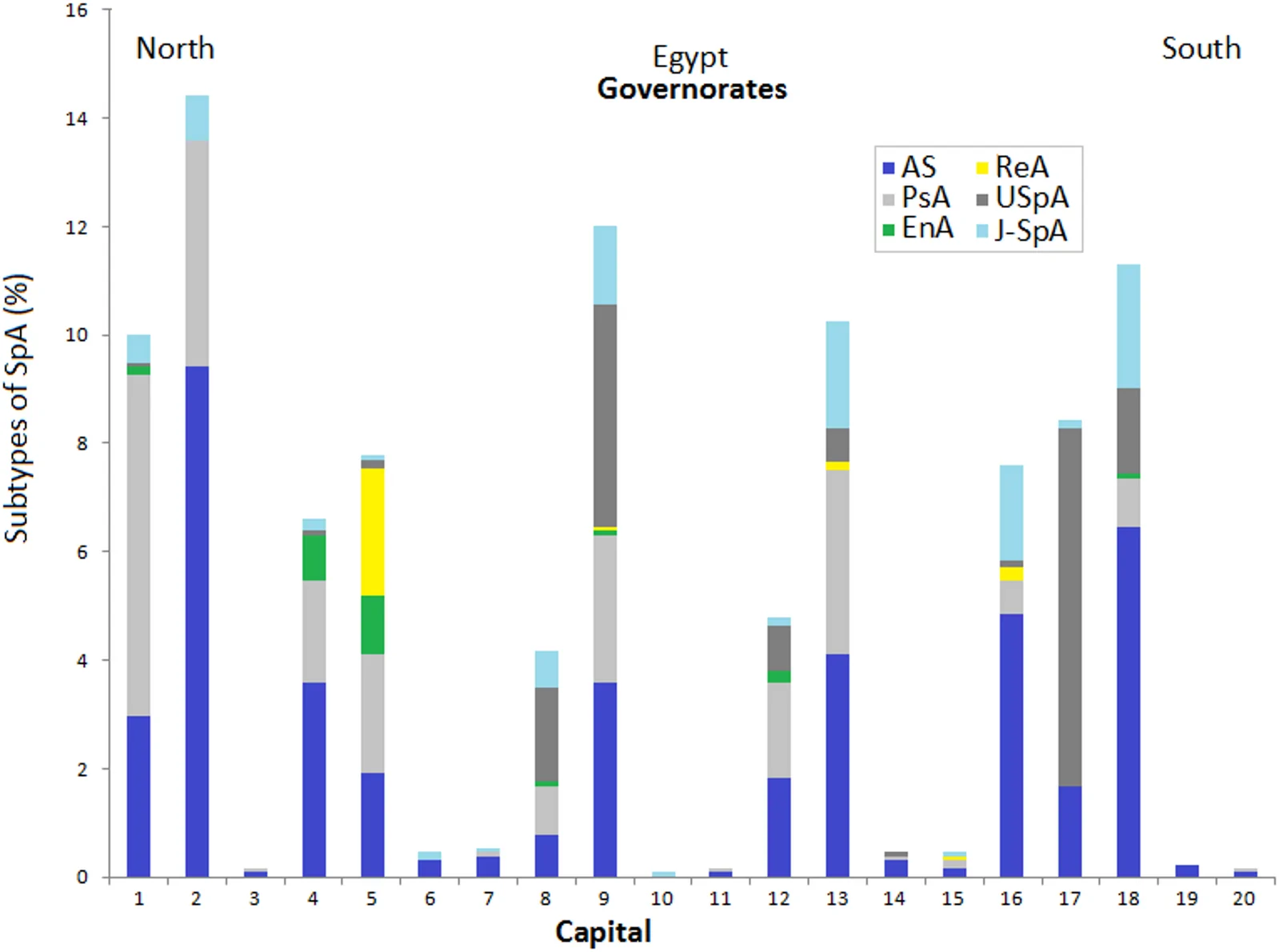
Subtypes of spondyloarthritis across the various governorates in Egypt. 1: Alexandria, 2: Kafr ElSheikh, 3: Beheira, 4: Mansoura, 5: Gharbia, 6: Sharkia, 7: Menoufiya, 8: Kalyoubia, 9: Cairo, 10: PortSaid, 11: Suez, 12: Ismailia, 13: Giza, 14: Fayoum, 15: Beni-Suef, 16: Minia, 17: Sohag, 18: Assuit, 19: Qena, 20: Aswan.

The r-axSpA was most reported from Assuit (15.9%), nr-axSpA from Cairo (24.5%), peripheral arthritis only from Giza (30.7%), and unclassified from Kafr El-Sheikh (33.9%) (*p* = 0.002). The subtype distribution of SpA across the nation is presented in Fig. 1. The gender distribution was comparable across the country (*p* = 0.34). The age at onset was highest in Ismailia (37.03 ± 12.4 years) (*p* = 0.003). The highest BASDAI was reported from Menoufiya (5.9 ± 2.2) (*p* = 0.002), and HLAB27 positivity was highest from Kafr El-Sheikh (40.6%) (*p* = 0.001).

## Discussion

The European Spondyloarthropathy Study Group (ESSG) has proposed classification criteria that identify five major subtypes of SpA. Among these subtypes are undifferentiated spondyloarthritis (uSpA), PsA, reactive arthritis (ReA), AS, and arthritis linked to IBD (SpA-IBD)^23^. Early in this century, the idea that axSpA is a single disease with two subsets; r-axSpA and nr-axSpA, was introduced. The ASAS classification criteria for axSpA have been in effect since 2009 and in addition to traditional X-rays; changes in the SIJ identified by MRI and the identification of HLA B27 were included in the broad classification^4^. An early diagnosis is imperative in order to provide a timely opportunity, especially with the presence of effective treatment options like tumour necrosis factor (TNF) blockers^24^.

Understanding the complex overlapping features in SpA is not only crucial for a definitive diagnosis but also paves the way for precise prognostic and therapeutic implications. Interestingly, the mounting evidence of subclinical autoimmune gut inflammation in classified and unclassified SpA warrants special considerations to select the optimum lines of treatment^25^. Even though, etanercept has shown promising results in AS, both with and without radiographic sacroiliitis, as well as other symptoms of the illness such as PSO, enthesitis, and peripheral arthritis, it is not helpful in treating IBD, with limited effectiveness for uveitis. Furthermore, IL-17 A inhibitors are potentially successful in treating axSpA and PsA but not for uveitis. In light of this information, the Egyptian College of Rheumatology conducted a nationwide study to look at the broad range of clinical manifestations and disease characteristics among Egyptian patients in this domain.

In *males*, the age at onset was significantly lower, while r-axSpA, neuropsychiatric and pulmonary manifestations, HLA-B27 positivity, and receiving biologic therapy were significantly higher. In *females*, cutaneous manifestations and arthritis were significantly more frequent. Similarly, in a study from Argentina, the frequency of women with axSpA was higher and they had less radiographic compromise^26^. There could be a delay in diagnosis in women due to gender-specific variations in how axSpA manifests. The diagnostic performance of structural markers of spinal inflammation by MRI, such as ankylosis, erosion, sclerosis, fat metaplasia, and bone marrow edema (BME), are substantially lower in female patients with axSpA, while active inflammatory lesions show comparable performance in both sexes, while still overall inferior to structural markers. This leads to a comparably higher risk of false positive findings in women^27^.

The prevalence of HLA-B27 and the incidence of axSpA are intricately connected and exhibit remarkable variation across the globe. Africa stands out with the lowest prevalence, whereas Northern Europe and North America boast the highest rates^28^. Unfortunately, specific data for Egypt remains elusive. In this study, the frequency of HLA-B27 in Egypt ranged widely from 1 to 33%, depending on the subtype.

Those with positive *HLA-B27* were of a significantly higher frequency in the AS subtype (73%) (*p* = 0.003), male gender (66.2%) (*p* < 0.0001), longer disease duration (*p* = 0.001), and were receiving a higher frequency of biologic therapy (89%) (*p* < 0.0001). HLA-B27 positivity is associated with a classic axSpA pattern worldwide, and its absence is associated with peripheral manifestations and worse outcomes, suggesting a relevant phenotypic difference in a highly miscegenated population^29^. If nothing else, HLA-B27 plays an important role in the diagnosis, classification, and determination of the severity of axSpA^30^. The strong genetic association between HLA-B27 and SpA accounts for 90% of the susceptibility to axSpA. However, as there are cases with negative HLA-B27, other HLA alleles, beyond HLA-B27, could be useful in facilitating SpA diagnosis, particularly in patients with a clinical picture consistent with SpA but not fulfilling the ASAS classification criteria^31^.

In relation to *subtypes*, PsA patients were more often female and had a higher frequency of fibromyalgia. Despite comparable disease characteristics, it has been reported that women with PsA have reduced treatment response to their first TNFi, highlighting the need to consider the gender in PsA research and management^32^.

Fibromyalgia might emerge in SpA, and it has been projected to have a prevalence ranging from 11to 25% [6]. In the present study, the frequencies of fibromyalgia varied according to the subtypes. The intersection of fibromyalgia with enthesitis, a hallmark of axSpA, underscores the complexity of the differential diagnosis^33^.

*Juvenile SpA* cases had the highest frequency of enthesitis. The late diagnoses of SpA, hip and spine involvement, with higher frequency of biologic treatment, were reported in juvenile-onset SpA^34^. Patients with *undifferentiated SpA* had a higher frequency of nr-axSpA and received more biologic therapy. Patients with ‘axSpA at risk’ show worse self-reported outcomes over time and are less likely to benefit from anti-inflammatory treatment than those with a classical axSpA phenotype^35^.

In relation to *radiological features*, in this work, r-SpA was more frequent in those with AS. AS was more common in males, and had a higher frequency of HLA-B27 with high disease activity and physical dysfunction. It has been reported that patients with r-axSpA were mainly males, with a younger age at onset, a higher prevalence of HLA-B27, more uveitis and enthesitis, fewer episodes of dactylitis, and higher disease activity than nr-axSpA^36^.

In relation to the *geolocation* across the country, the r-axSpA was most reported from Assuit (15.9%), the nr-axSpA from Cairo (24.5%), and peripheral arthritis only from Giza (30.7%), and the unclassified SpA from Kafr El-Sheikh (33.9%). The age at onset was highest in Ismailia, the BASDAI was reported higher in Menoufiya and HLAB-27 (*p* = 0.001) positivity was highest from Kafr El-Sheikh.

In this work, the *disease activity* was comparable between males and females with a rise in association to the disease subtype. It has been reported that the changes in disease activity before and after TNFi use were significantly different between sexes when measured by BASDAI, but not ASDAS^37^. Almost half the patients were active with a BASDAI score exceeding 4, while over 50% displayed inactive or mild disease. Notably, females exhibited higher disease activity than males. Females are known to have a less favorable response to anti-TNF treatment^38^.

Currently, p*hysical dysfunction* was comparable according to the gender but increased in those with AS. Spinal damage is independently associated with physical function in axSpA and no difference in BASFI was found according to gender in r-axSpA^13^.

Recent literature underscores the complexity and challenge of achieving remission in patients with axSpA. Reported remission rates vary widely across cohorts and clinical trials but are generally low in real-world practice, ranging from as low as 0.6% in our cohort to about 20–40% in other clinical series^1,2^. Factors consistently associated with higher likelihood of remission include younger age, male gender, lower baseline disease activity and functional impairment, positive HLA-B27 status, and early initiation of disease-modifying therapies particularly biologic agents such as TNF inhibitors^41^. Conversely, delays in diagnosis and treatment are strongly correlated with worse clinical outcomes, reduced response to therapy, and lower rates of remission^42^. Socioeconomic disparities, limitled access to advanced therapies, and suboptimal adherence also negatively impact remission rates^40^.

Among patients receiving biologic therapy in our cohort, secukinumab was the most frequently prescribed agent (34.4%), followed by adalimumab (31.9%), etanercept (20.5%), and other biologics. This frequent use of secukinumab aligns with recent evidence supporting its rapid and sustained efficacy in reducing disease activity, its favorable safety profile, and high patient retention in both clinical trials and real-world settings^43^. Secukinumab, an interleukin-17 A inhibitor, has demonstrated robust benefits for both r- and nr- axSPA and is now endorsed as a first-line or alternative biologic by international guidelines, especially for patients who are intolerant or have inadequate response to tumor necrosis factor inhibitors (TNFi)^44^. Additionally, practical factors such as physician familiarity, patient preference, and local availability may further contribute to its predominance in routine clinical practice in Egypt^45^.

In the present work, while conventional *treatment* was more received by females, biologic therapy was more used in males and in those with AS or undifferentiated SpA. Females appear more likely to continue on NSAIDs after diagnosis, and the time to initiation of bDMARDs was longer for females than males^46^.

In the present study, the mean age at onset of the SpA cases was 29.9 years; with a M: F 1.4:1 and 19.8% had HLA-B27 positivity. On the contrary, in a large cohort Chinese study, the median age at disease onset of nr-axSpA was 22 years. M: F was 1.26:1 and the HLA-B27 positivity was 72.2%^47^,.

Considering a longitudinal study design in future work may help in understanding more the varied faces of SpA and diverse landscape.

A major strength of this study is its unprecedented scale, representing the largest multicenter cohort of SpA patients conducted to date in Egypt and the Middle East. The inclusion of 1,401 patients from 15 specialized rheumatology centers spanning 20 major cities ensures thorough geographic and demographic coverage, which strengthens the generalizability of the findings to the Egyptian SpA population and offers valuable insight into regional disparities. However, several limitations must be noted. The cross-sectional design precludes analysis of longitudinal outcomes. Patient recruitment was uneven across centers, resulting in potential regional selection bias and impacting the generalizability of findings regarding subtype prevalence and HLA-B27 frequency. The lack of centralized imaging review allowed radiographic and MRI interpretations at individual centers, introducing variability in the classification of r-axSpA and nr-axSpA subtypes. Primary reliance on univariate analyses, without multivariable adjustment for confounding factors such as age, sex, and disease duration, limits the robustness of observed associations. These methodological considerations should be carefully weighed in interpreting the results and their implications for SpA epidemiology and management in Egypt.

## Conclusion

The spectrum of SpA in Egypt is inconsistent across the country. Gender, disease subtype and HLA-B27 seem to play a key role in the phenotypic presentation.

## Data Availability

The datasets used and/or analysed during the current study are available from the corresponding author on reasonable request.
